# Ipiq-Enlil and the Early Development of Veterinary Specialisation: Reassessing the Mari Letter from the Perspective of Veterinary History

**DOI:** 10.3390/vetsci13090903

**Published:** 2026-09-03

**Authors:** Agata Małyszek, Maciej Janeczek

**Affiliations:** Department of Biostructure and Animal Physiology, Wrocław University of Environmental and Life Sciences, C.K. Norwida 31, 50-375 Wrocław, Poland; agata.malyszek@upwr.edu.pl

**Keywords:** ancient Mesopotamia, animal healing, cuneiform texts, history of veterinary medicine, Ipiq-Enlil, Mari, One Health, veterinary specialisation

## Abstract

The origins of veterinary medicine remain a subject of debate. The search for the “first veterinarian” has often led to the application of modern professional concepts to ancient societies. Among the Mesopotamian sources, particular attention has been given to ARM XXVI/1, text 270 from the royal archives of Mari, in which Jean-Marie Durand described Ipiq-Enlil as une espèce de vétérinaire (“a kind of veterinarian”). Despite the potential importance of this interpretation for the history of veterinary medicine, it has never been critically examined in the veterinary literature. The present study reassesses the letter concerning Ipiq-Enlil in the light of Durand’s philological commentary and compares it with other key Near Eastern sources relating to animal healthcare, including the Kahun Veterinary Papyrus, §§224–225 of the Code of Hammurabi, the records concerning Abū-ilīšu, and the seal of Ur-lugal-edinna. Our analysis indicates that the expression *une espèce de vétérinaire* represents Durand’s philological interpretation rather than a literal translation of the cuneiform text. Comparison with other Near Eastern sources shows that practical knowledge relating to animal health was already well developed during the first half of the second millennium BCE. At the same time, the available evidence does not support the existence of a distinct veterinary profession in the modern sense. Instead, the case of Ipiq-Enlil points to an early stage in the differentiation of specialised animal healthcare from a shared medical tradition encompassing both humans and animals.

## Simple Summary

The origins of veterinary medicine are still not fully understood because ancient sources rarely describe the people who treated sick animals. For many years, historians have attempted to identify the “first veterinarian”, but this approach may project modern professional concepts onto ancient societies. In this study, we re-examined a royal letter from the ancient Mesopotamian city of Mari, written approximately 3800 years ago, concerning an official named Ipiq-Enlil. Although he has previously been described as “a kind of veterinarian”, this interpretation has never been critically evaluated from the perspective of veterinary history. By comparing this letter with other sources from Mesopotamia and Egypt, we show that it provides important evidence for the gradual emergence of specialised knowledge and practice related to the treatment of animals. These findings help explain how specialised animal healthcare emerged and show that the close relationship between human and animal medicine has deep historical roots.

## 1. Introduction

Despite decades of research, the origins of veterinary medicine remain incompletely understood, particularly with regard to the emergence of animal treatment. Early studies focused primarily on identifying the earliest evidence of animal healing and on determining who might be regarded as the first practitioners responsible for the treatment of animals [1,2,3]. Consequently, figures such as Ur-lugal-edinna and Abū-ilīšu have frequently been presented as the earliest known specialists associated with veterinary practice [4,5,6]. More recently, however, this approach has been questioned because it risks projecting modern professional concepts onto societies in which clearly defined occupational boundaries had not yet developed. Rather than searching for the “first veterinarian”, recent research has increasingly focused on understanding how specialised knowledge and practice relating to the treatment of animals gradually emerged from a broader medical tradition shared by humans and animals [7,8,9]. The royal archives of Mari provide an exceptional opportunity to explore this process. Although these texts have long been recognised as one of the principal sources for the history of ancient Mesopotamia, they have rarely been examined from the perspective of veterinary history. They provide valuable information on the organisation of animal care and on the relationship between animal treatment and the broader medical traditions of the ancient Near East [10,11,12]. Among these texts, the letter concerning Ipiq-Enlil deserves particular attention. In his philological commentary, Jean-Marie Durand argued that the terminology employed in the document referred to an individual whose expertise extended beyond that of an ordinary animal caretaker. He therefore described Ipiq-Enlil as *une espèce de vétérinaire* (“a kind of veterinarian”) [11]. Although potentially important for the history of veterinary medicine, this interpretation has received remarkably little attention outside Assyriology. Since its publication, it has not been critically evaluated alongside other key evidence relating to the early development of animal treatment, including the Kahun Veterinary Papyrus [13,14], §§224-225 of the Code of Hammurabi [15], the records concerning Abū-ilīšu, and the seal of Ur-lugal-edinna [1,3].

Building on Durand’s philological commentary, this study examines the Mari letter from the perspective of veterinary history. To our knowledge, this is the first study in the veterinary literature to evaluate the significance of Durand’s proposal within the broader context of the emergence of veterinary specialisation in the ancient Near East. Our aim is to assess the significance of this document for understanding the early development of specialised knowledge and practice relating to the treatment of animals.

## 2. Materials and Methods

This study is based on cuneiform texts from the royal archives of Mari dating to the eighteenth century BCE. It combines philological analysis with a comparative historical approach to reassess the significance of Ipiq-Enlil for the early development of specialised animal healthcare. The principal source was ARM XXVI/1, text 270 (A.2355), published by Jean-Marie Durand in the first volume of the *Archives Royales de Mari* series [11]. The document was selected because it contains the only known reference to Ipiq-Enlil interpreted as describing an individual whose expertise extended beyond routine animal care. Durand’s critical edition, including the transliteration, translation, and philological commentary, formed the basis of the analysis. The Mari letter was also considered in the context of other published correspondence from the archive dealing with animal care, herd management, and the treatment of animals.

Comparative sources were selected because they provide complementary evidence for specialised animal healthcare in the ancient Near East. Primary sources were included when they documented therapeutic practice, legal regulation, administrative responsibilities, or professional roles connected with animal care. These comprised the Kahun Veterinary Papyrus [13,14], the relevant provisions of the Code of Hammurabi [15], the evidence concerning Abū-ilīšu [3], and the seal of Ur-lugal-edinna [1]. The comparative analysis considered the terminology used for individuals involved in animal care, the historical and administrative context of each source, the animals concerned, and the nature of the activities described. Particular attention was given to whether the evidence reflected routine animal management, therapeutic activity, or a more specialised form of animal healthcare. The sources were compared in terms of their contribution to understanding the gradual differentiation of specialised animal care rather than as direct textual parallels.

Published studies in Assyriology and the history of veterinary medicine were consulted to evaluate earlier interpretations and to place the findings within their broader historical context. Primary sources formed the basis of the analysis, whereas secondary literature served to support philological interpretation and historical contextualisation.

The manuscript was originally written in Polish and translated into English by the authors using DeepL Translator. During the preparation of the English version, ChatGPT (OpenAI, GPT-5 model family) was also used for linguistic proofreading, to organise selected sections of the text, and to check the formatting of citations and the reference list. These tools were not used to generate original data or figures. The selection of sources, historical and philological interpretations, verification of the bibliography and the final wording of the text have been checked and approved by the authors.

## 3. Results

The royal archives of Mari are among the most important written sources for the history of the ancient Near East. Discovered during the French excavations at Tell Hariri, which began in 1933, they contain approximately 20,000 cuneiform tablets and fragments dating mainly to the reigns of Yasmah-Addu and Zimri-Lim in the eighteenth century BCE [10,16]. Administrative letters form the largest part of this corpus, supplemented by economic, legal, and accounting documents. Unlike normative or literary texts, these records were produced as part of the daily administration of the kingdom and document matters relating to estate management, the royal court, official activities, and the breeding, transport, and care of domestic animals [10,17]. As a result, they provide valuable insight not only into animal husbandry but also into the organisation of responsibilities associated with animal care within the royal administration. The principal source analysed in this study is ARM XXVI/1, text 270 (A.2355), published by Jean-Marie Durand in the first volume of the *Archives Royales de Mari* series [11]. Written by Tarim-Šakim to King Yasmah-Addu, it concerns Ipiq-Enlil, an official who had previously served under Samsî-Addu. Rather than recording an administrative decision, the text takes the form of a recommendation in which the sender reminds the king of Ipiq-Enlil’s abilities, emphasizes his value to the royal administration, and concludes by stating that “this man is capable of everything” [11]. Despite its brevity, the document contains the passage that forms the basis of the present analysis.

The relevant section (lines 6–17), translated after Durand [11], reads as follows:

“…Once or twice already I have spoken to my lord about Ipiq-Enlil. This man knows perfectly well how to look after animals and act as a physician. My lord should pay attention to him. I have requested this man from the king. This man is entirely competent. My lord should not dismiss him…”

No information is provided about Ipiq-Enlil’s official duties or everyday responsibilities. Instead, the emphasis falls on his abilities and the confidence placed in him by the author of the letter. Particularly striking is the explicit association between caring for animals and acting as a physician. Because the document offers no further explanation of these responsibilities, understanding its significance depends largely on Durand’s philological interpretation. A central element of Durand’s interpretation is the functional term *kīzum*. In line 8, the term appears in the form *ki-[iz]-tam*. In Assyriological scholarship, *kīzum* is generally understood as an administrative title, usually translated as “groom” or “stableman” and primarily associated with horse management. The title appears to have referred to an administrative position within the royal household rather than to an independent medical profession. Durand argued that the context of ARM XXVI/1, text 270 points to a broader range of responsibilities. Drawing on comparative cuneiform evidence, he noted that a *kīzum* could also have been responsible for sheep, donkeys and other domestic animals, implying duties that extended beyond ordinary animal husbandry. He further argued that the wording of the letter indicates that Ipiq-Enlil’s expertise extended to both animals and humans (*Le contexte actuel semble indiquer que l’habileté d’Ipiq-Enlil s’étend aussi bien aux animaux qu’aux humains*—“The present context seems to indicate that Ipiq-Enlil’s skills extended to both animals and humans”) [11]. These observations led Durand to describe Ipiq-Enlil as *une espèce de vétérinaire* (“a kind of veterinarian”). Durand also referred to §224 of the Code of Hammurabi, which regulates the fee paid to an *asû*, an Akkadian term commonly translated as “physician” or “healer”, for treating oxen and donkeys. The following provision, §225, addresses the responsibility of the practitioner when such treatment results in the death of the animal [15]. The expression, however, is clearly interpretative rather than a literal translation of the cuneiform text and deliberately avoids equating Ipiq-Enlil with the modern concept of a veterinarian. The remaining correspondence from Mari shows that matters concerning animals formed an important part of royal administration. Numerous letters deal with herds, the transport of animals, supplies, and their maintenance, whereas references to animal diseases or to individuals responsible for treating sick animals are comparatively uncommon. ARM XXVI/1, text 271 provides an instructive comparison. It reports the death of dogs in the Bisri region from a disease interpreted by the editor as rabies [11]. Rabies and rabid dogs are also attested in other Mesopotamian legal, medical, literary, and magical texts [18]. Although the letter shows that animal disease outbreaks were reported to the royal administration, it says nothing about treatment or about the individuals responsible for the animals’ care. In this respect, ARM XXVI/1, text 270 is exceptional within the Mari correspondence because it focuses on a named individual whose expertise combined animal care with medical knowledge. Among the currently known texts from Mari, it remains the only document that explicitly associates expertise in animal care with medical competence in the same individual, making it one of the most informative surviving sources for understanding the early development of specialised knowledge relating to the treatment of animals in Old Babylonian Mesopotamia.

**Figure 1:**
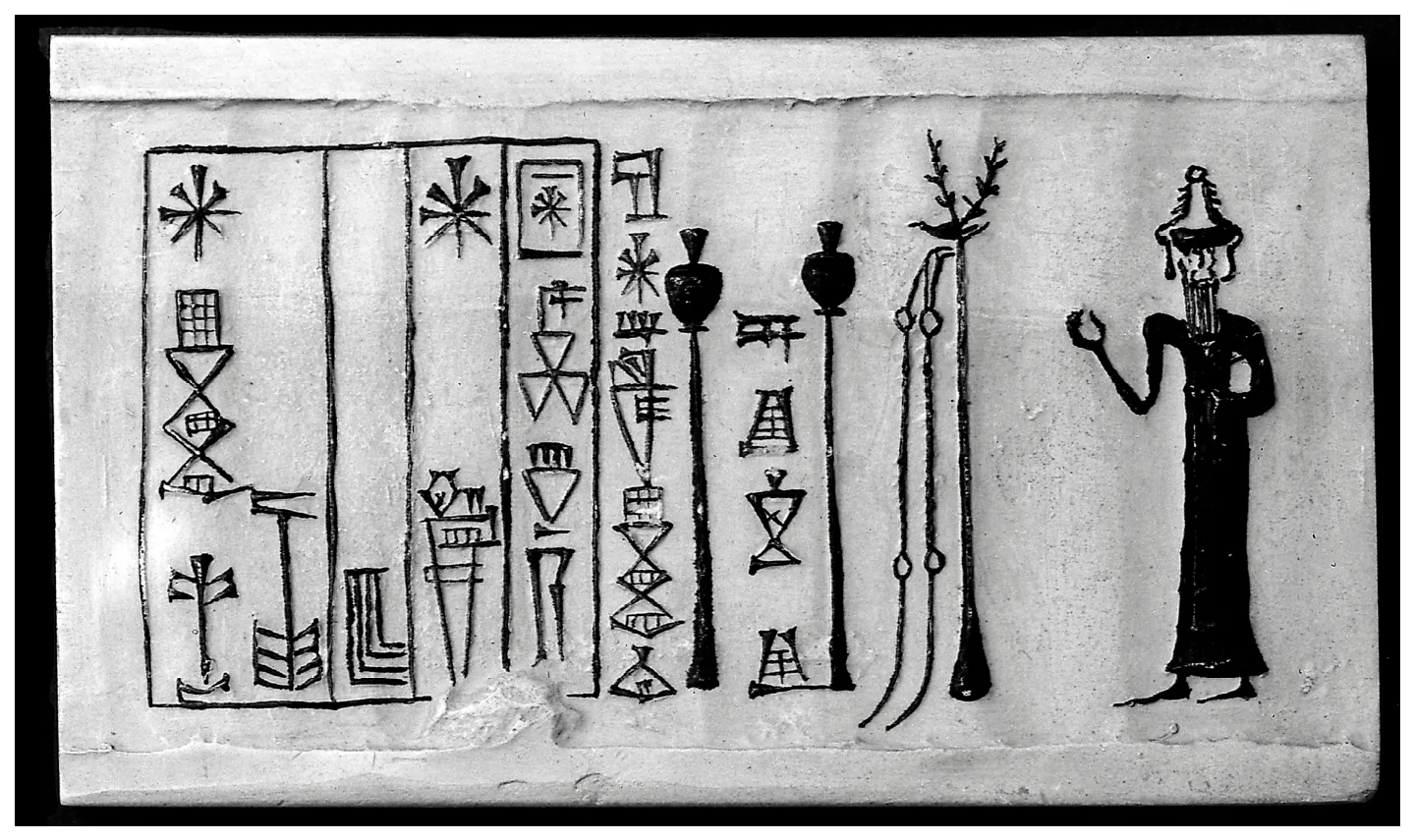
Impression of the cylinder seal of Ur-lugal-edinna (mid-third millennium BCE). Fuhr [1] interpreted the combined inscription and iconography of the seal as evidence of specialised involvement in animal healthcare. Image credit: Wellcome Collection (M0014664), CC BY 4.0.

The principal Near Eastern sources discussed in this study are compared in Table 1, and the cylinder seal of Ur-lugal-edinna is shown in Figure 1.

## 4. Discussion

In his commentary on ARM XXVI/1, text 270, Jean-Marie Durand described Ipiq-Enlil as une espèce de vétérinaire (“a kind of veterinarian”) [11]. Although well known in Assyriological scholarship, this interpretation has received little attention in the veterinary literature [1,2,3,4,5,6,11,19]. Durand was careful not to describe Ipiq-Enlil simply as a *vétérinaire*. By choosing the expression *une espèce de vétérinaire*, he made it clear that he was proposing a philological interpretation rather than translating an explicit professional title preserved in the cuneiform text [11]. This distinction is important because the Mari letter neither defines Ipiq-Enlil’s official duties nor records any diagnostic or therapeutic procedures. Instead, it presents him as an individual whose expertise combined responsibility for animals with medical knowledge and whose abilities were highly valued within the royal administration. The letter points to a stage at which knowledge relating to animals had become associated with medical expertise in the same individual, without implying the existence of an independent veterinary profession. It therefore provides evidence for an intermediate phase in the development of specialised animal healthcare rather than for a profession already recognised as distinct [7,9].

When considered alongside other early Near Eastern evidence, this interpretation gains additional support. The Kahun Veterinary Papyrus demonstrates that sophisticated diagnostic and therapeutic procedures were already in use [13,14,20,21], whereas §§224–225 of the Code of Hammurabi show that the treatment of animals was subject to legal regulation, including both remuneration and practitioner liability [15]. References to Abū-ilīšu likewise indicate the existence of specialists associated with animal medicine, although the precise scope of their duties remains uncertain [3]. Evidence associated with Ur-lugal-edinna has also been linked with specialised animal healthcare, although this interpretation is based on a combined reading of the seal inscription and its iconography (Figure 1) [1]. The inscription invokes Edin-mu-gi, the servant of the god Gir, a deity associated with herds and the protection of animals, particularly their reproduction, and includes an epithet referring to assistance during childbirth. In 1966, Fuhr interpreted the two objects suspended from the branched pole as obstetrical ropes used to assist difficult deliveries in animals [1]. Similar obstetrical ropes are depicted in ancient Egyptian reliefs illustrating assisted calving, indicating that such techniques were recognised elsewhere in the ancient Near East [4,5]. None of these sources, however, documents a clearly defined veterinary profession. Instead, they indicate the gradual differentiation of specialised animal healthcare from a broader medical tradition shared by humans and animals [7,19,20,22].

Within this wider historical context, the Mari letter occupies a distinctive position. Unlike legal texts or therapeutic manuals, it focuses on a named individual and explicitly associates his expertise with both animals and medicine. This makes it a valuable source for understanding the place of animal healthcare within the administrative structures of Old Babylonian Mesopotamia [11].

The Mari letter does not demonstrate the existence of an early veterinary profession. Instead, it broadens the documentary basis for studying the origins of veterinary specialisation by bringing an overlooked administrative source into veterinary historiography. Considered alongside other Near Eastern evidence, it offers a new perspective on how specialised animal healthcare gradually emerged from a broader medical tradition shared by humans and animals.

### Limitations of the Evidence

The evidence provided by ARM XXVI/1, text 270 must be interpreted with caution because of the nature of the surviving source. The document is a short letter of recommendation rather than an administrative or medical record, and it was not intended to describe either the scope of Ipiq-Enlil’s responsibilities or the procedures he performed. This probably reflects the fact that both the sender and the recipient were already familiar with his duties, making further explanation unnecessary. Consequently, only limited information is available, and its significance can be assessed through philological analysis and comparison with other Near Eastern sources [11].

The philological basis of the study also needs to be taken into account. Our analysis follows Durand’s published edition, transliteration, translation, and commentary, and does not involve an independent retranslation of the original cuneiform text. The focus here is therefore on the historical significance of Durand’s interpretation rather than on proposing a new reading of the document.

Another limitation is the absence of additional documents referring directly to Ipiq-Enlil. No surviving text describes his daily activities, the methods he used, or his precise position within the royal administration. Any reconstruction of his responsibilities therefore depends on a single source interpreted alongside comparative evidence from Mesopotamia and Egypt [9,23]. As Finkel and Geller [22] observed, modern professional categories cannot be applied directly to cuneiform sources. Terms such as veterinarian are modern constructs and have no exact equivalents in the textual record of the second millennium BCE. Durand’s expression *une espèce de vétérinaire* should therefore be regarded as a cautious philological interpretation rather than as evidence for a formally recognised profession [11].

These limitations define the scope of the conclusions that can be drawn from the Mari letter. Rather than identifying the earliest veterinarian, the document provides further evidence of this transitional stage in the development of animal healthcare.

## 5. Conclusions

The administrative archives from Mari are an important source for the history of veterinary medicine because they document one of the earliest stages in the development of specialised animal healthcare. ARM XXVI/1, text 270 does not describe therapeutic procedures. Its importance lies in its portrayal of an individual whose expertise extended beyond routine animal care and included medical knowledge.

Our reassessment of Jean-Marie Durand’s philological commentary suggests that the expression *une espèce de vétérinaire* is an interpretative conclusion rather than a translation of the cuneiform text. Although this interpretation has been recognised in Assyriological scholarship for decades, it has received little attention in the veterinary literature. Considered together with other early Near Eastern evidence, the Mari letter provides a broader historical context for the emergence of veterinary expertise.

The available sources indicate that practical knowledge relating to animal health was already well developed during the first half of the second millennium BCE. At the same time, they provide no evidence for the existence of an independent veterinary profession in the modern sense. Instead, the case of Ipiq-Enlil illustrates an early stage in the differentiation of specialised animal healthcare from a shared medical tradition encompassing both humans and animals. Our findings also suggest that reconstructing the early history of veterinary medicine requires evidence drawn from administrative, legal, and medical sources. This shared medical tradition should not be regarded as an early form of the modern One Health concept. Rather, it reflects a period before human and animal medicine developed into separate fields of knowledge and professional practice.

## Figures and Tables

**Table 1:** Comparison of the principal Near Eastern sources discussed in this study.

Source	Type of Source	Date and Region	Ancient Terminology	Evidence Related to Animal Healthcare	Historical Interpretation
Mari letter (ARM XXVI/1, text 270)	administrative letter	18th century BCE, Mari	*kīzum*, *asû*	Named individual combining responsibility for animals with medical expertise	Early differentiation of specialised animal healthcare
Mari letter (ARM XXVI/1, text 271)	administrative letter	18th century BCE, Mari/Bisri region	_	Report of deaths among dogs from a disease interpreted as rabies	Administrative reporting of animal disease, without evidence of treatment or specialised animal healthcare
Kahun Veterinary Papyrus	veterinary papyrus	c. 1850–1800 BCE, Egypt	_	Diagnostic and therapeutic procedures for cattle	Advanced animal healthcare; no evidence of a distinct veterinary profession
Code of Hammurabi (§§224– 225)	law code	18th century BCE, Babylon	*asû*	Legal regulation of treatment of oxen and donkeys	Legal recognition of animal medical practice
Administrative text concerning Abū-ilīšu	administrative text	Old Babylonian Mesopotamia	*asû alpī*	Specialist explicitly associated with cattle medicine	Evidence for specialised animal healthcare; exact duties remain uncertain
Cylinder seal of Ur-lugal-edinna	cylinder seal	Mid-third millennium BCE, Sumer	*a-zu*	Combined epigraphic and iconographic evidence associated with animal healthcare	Possible specialised involvement in animal healthcare

## Data Availability

No new datasets were generated during this study. All data analysed are contained within this article and the cited published primary and secondary sources.
